# Causes behind error rates for predictive biomarker testing: the utility of sending post-EQA surveys

**DOI:** 10.1007/s00428-020-02966-7

**Published:** 2020-11-23

**Authors:** Cleo Keppens, Ed Schuuring, Elisabeth M. C. Dequeker

**Affiliations:** 1 Department of Public Health and Primary Care, Biomedical Quality Assurance Research Unit, University of Leuven, Kapucijnenvoer 35 block d, 1st floor, box 7001, 3000 Leuven, Belgium; 2 Department of Pathology, University of Groningen, University Medical Center Groningen (UMCG), (HPC EA10), Hanzeplein 1, PO Box 30001, 9700 RB Groningen, The Netherlands

**Keywords:** External quality assessment, Molecular pathology, Root cause analysis, Quality management, Biomarkers, ISO 15189, Colorectal cancer, Non-small-cell lung cancer

## Abstract

External quality assessment (EQA) schemes assess the performance of predictive biomarker testing in lung and colorectal cancer and have previously demonstrated variable error rates. No information is currently available on the underlying causes of incorrect EQA results in the laboratories. Participants in EQA schemes by the European Society of Pathology between 2014 and 2018 for lung and colorectal cancer were contacted to complete a survey if they had at least one analysis error or test failure in the provided cases. Of the 791 surveys that were sent, 325 were completed including data from 185 unique laboratories on 514 incorrectly analyzed or failed cases. For the digital cases and immunohistochemistry, the majority of errors were interpretation-related. For fluorescence in situ hybridization, problems with the EQA materials were reported frequently. For variant analysis, the causes were mainly methodological for lung cancer but variable for colorectal cancer. Post-analytical (clerical and interpretation) errors were more likely detected after release of the EQA results compared to pre-analytical and analytical issues. Accredited laboratories encountered fewer reagent problems and more often responded to the survey. A recent change in test methodology resulted in method-related problems. Testing more samples annually introduced personnel errors and lead to a lower performance in future schemes. Participation to quality improvement projects is important to reduce deviating test results in laboratories, as the different error causes differently affect the test performance. EQA providers could benefit from requesting root cause analyses behind errors to offer even more tailored feedback, subschemes, and cases.

**Supplementary Information:**

The online version contains supplementary material available at 10.1007/s00428-020-02966-7.

## Introduction

The analysis of tumor-specific biomarkers provides information for appropriate targeted treatment decision-making in non-small-cell lung cancer (NSCLC) and metastatic colorectal cancer (mCRC) [1–3]. Predictive biomarker test results should therefore be accurate, reproducible and timely.

Several external quality assessment (EQA) schemes, organized on a national or international level, assessed the performance for common biomarkers in NSCLC and mCRC. They revealed varying error rates depending on the evaluated markers and variants, sample types, or scheme rounds [4–13].

Longitudinal analyses of the EQA schemes organized by the European Society of Pathology (ESP) revealed that participation to multiple EQA scheme rounds improved participants’ performances [12, 13]. Over time, error rates decreased for *ALK* and *EGFR* analysis but increased for *ROS1*. Also, error rates were higher for immunohistochemistry (IHC) compared to fluorescence in situ hybridization (FISH) on formalin-fixed paraffin embedded (FFPE) samples and especially compared to digital case interpretation [12]. Remarkably, lower error rates have been described for cell lines compared to resections, for higher variant allele frequencies [13], and for laboratories who are accredited, test more samples or perform research [14]. In mCRC, error rates increased significantly for mutation-positive samples and for methods that do not cover all required variants [11].

Medical laboratories are advised to participate in EQA schemes [1, 3] sometimes part of their quality framework conform the International Organization for Standardization (ISO) standard 15189:2012 [15] or national equivalents like CAP 15189 [16]. Laboratories should have a documented procedure to identify and manage non-conformities when pre-determined performance criteria are not met, both for EQA as in routine practice.

The providers of these EQA programs are preferably accredited according to ISO 17043:2010 [17], mimic patient samples as closely as possible, and check the entire examination process [15]. EQA providers could guide laboratories by the provision of feedback, reference material, or methodological advice [18, 19]. Some providers (such as the CAP and UK NEQAS) already request a root cause analysis from poor performers [7, 15], but no data has yet been published. Errors can be systematic (e.g., test method failure) while others can be accidental (e.g., clerical or pipetting errors). The time point of error occurrence in the total test process (TTP) has been reported in clinical chemistry and forensics [20, 21] and were mostly pre- (46–86%) and post-analytical (18–47%) of nature [20]. However, data is still lacking for molecular oncology.

Recently, a step-by-step framework for effective EQA results management was proposed for laboratories and EQA providers [22, 23]. A subsequent evaluation of deviating EQA results in clinical chemistry according to this flowchart revealed that most errors (81%) were the laboratory’s responsibility (internal causes) and were mainly clerical errors (i.e., correct outcome entered incorrectly in the results form) (72%) [22].

This study evaluated the feasibility of requesting root causes of deviating EQA results in the ESP schemes for NSCLC and mCRC between 2014 and 2018. The error causes were compared for the different markers, techniques, and sample types, as well as for different laboratory characteristics.

## Material and methods

The ESP schemes were organized according to the requirements for EQA programs in molecular pathology [18] and ISO 17043 [17]. Laboratories could register to several subschemes for different techniques and markers. Sample selection and preparation, validation by the reference laboratories, and distribution to participants were previously described [11, 12]. Laboratories received 14 calendar days to analyze all samples by their routine methodology and return an electronic datasheet on the cases’ outcomes, the applied test methodology, and laboratory characteristics. Reported laboratory settings and accreditation statuses were further validated on the websites of the laboratories and national accreditation bodies, respectively. The correctness of the sample outcomes was assessed by a team of international experts according to predefined scoring criteria [11, 12]. Participants received feedback including a general scheme report, participation certificate, and individual comments.

At the end of the EQA schemes, laboratories with at least one error or analysis failure (i.e., no outcome was available due to a test failure) were invited via e-mail to complete a survey with case-specific questions for every incorrect or failed case. The total number of participants and cases analyzed is summarized in Table 1. The survey was drafted in Microsoft Excel Developer and tailored to the participants’ own results **(**Supplemental Data 1**)**. This information included the case number and the type of deviation from the validated outcome for every subscheme (false-positive or false-negative results, variant reported at an incorrect position or gene, or over- and underestimations of the tumor proportion score (TPS) for *PD-L1*). Questions included pre-developed dropdown lists and checkboxes for ease of completion.

**Table 1:** Number of cases analyzed per subscheme offered in the ESP EQA schemes

Year			2015	2016	2017	2018	Study total
Analyzed laboratories	# EQA participations to different subschemes		329	445	733	712	2219
	# unique laboratories participating		197	234	259	241	410*
	# unique laboratories who received the survey (laboratories with at least one error in any of the subschemes)		88	120	153	175	315*
	# unique laboratories who replied to the survey		39	44	90	99	185*
Analyzed surveys	# of surveys sent		105	154	234	298	791
	# of survey responses received		40	53	108	124	325
Analyzed cases	# cases tested in the scheme		4224	5134	6276	5902	21,536
	# deviating EQA results included in survey		162	225	362	418	1167
	# deviating EQA results with response		51	74	181	208	514
# deviating EQA results with response for NSCLC	FISH digital	*ALK*	1	0	4	2	7
		*ROS1*	4	3	5	21	33
	IHC digital	PD-L1	N/A	N/A	11	19	30
	FISH	*ALK*	2	4	18	1	25
		*ROS1*	6	3	7	33	49
	IHC	ALK	0	4	20	7	31
		ROS1	9	0	2	6	17
		PD-L1	N/A	N/A	35	28	63
	IHC technical	ALK	8	6	7	15	36
		ROS1	N/A	1	0	2	3
		PD-L1	N/A	N/A	N/A	6	6
	Variant analysis	*EGFR* (mandatory)	21	19	41	27	108
		*KRAS* (optional)	N/A	N/A	2	11	13
		*BRAF* (optional)	N/A	N/A	0	3	3
# deviating EQA results with response for mCRC	Variant analysis**	*KRAS* (mandatory)	N/A	23	23	22	68
		*NRAS* (mandatory)	N/A	7	2	3	12
		*BRAF* (optional)	N/A	4	4	2	10

Laboratories were free to participate to one of the techniques for a selected marker. Participation to FISH digital was mandatory for the same marker if a laboratory registered for FISH for that marker, and participation to IHC digital or technical was mandatory for IHC participants for the same marker. *One unique laboratory could have participated, received the survey, and replied to the survey in several scheme years, which is why the total number of unique participants does not equal the sum of the different years. **WT cases and cases without neoplastic cells are included within the schemes for *KRAS*, *NRAS*, and *BRAF* to test the performance of the laboratories to denote these samples as a wild-type status or case without neoplastic cells

*N/A*, not applicable as no surveys were sent (no EQA scheme offered or only a pilot, or scheme outside the study period); *ALK*, ALK receptor tyrosine kinase; *BRAF*, B-Raf proto-oncogene; *EGFR*, epidermal growth factor receptor; *EQA*, external quality assessment; *ESP*, European Society of Pathology; *FISH*, fluorescence in situ hybridization; *IHC*, immunohistochemistry; *KRAS*, KRAS proto-oncogene; *mCRC*, metastatic colorectal cancer; *NRAS*, NRAS proto-oncogene; *NSCLC*, non-small cell lung cancer; *PD-L1*, programmed death ligand 1; *ROS1*, ROS proto-oncogene 1

Laboratories received additional information on the study set-up and a list of definitions on the applied terminology to harmonize responses for statistical analysis. The returned survey data were thereafter linked to the datasheet entries on laboratory setting and methodology during the EQA scheme, and the participants’ performances. The deadline for response was set at 1 month. Laboratories received a first reminder after 14 days and a second reminder the day before the deadline.

All survey responses from the ESP schemes for NSCLC between 2014 and 2018 and mCRC schemes between 2015 and 2018 were included. Statistics were performed using SAS software (version 9.4 of the SAS System for Windows, SAS Institute Inc., Cary, NC, USA). Statistical models with estimations using generalized estimating equations (GEE) were applied for clustering of identical laboratories participating to different schemes (NSCLC vs. mCRC) and years. Binary outcome variables were analyzed by logistic regression models. Ordinal and categorical outcome variables were analyzed by proportional odds models. Detailed statistics are shown in Supplemental Data 2.

## Results

### Response to root cause surveys

In the period between December 2015 and February 2019, 791 individual surveys were sent, to 315 unique laboratories from 43 countries. The probability of laboratories to receive the survey at the end of the EQA scheme (because they made an EQA error) and to respond to the survey is presented in Table 2 for the different laboratory characteristics.

**Table 2:** Probability of survey receipt and response for the different laboratory characteristics

Laboratory characteristics	*N*	Number of laboratories who received the survey (min 1 error in EQA scheme) (%)	Number of laboratories who responded (% of participants receiving the survey)
Setting (*n* = 2219)^a^	2219	791 (35.6)	325 (41.1)
Industry	71	33 (46.5)	15 (45.5)
(Private) laboratories	399	151 (37.8)	64 (42.4)
Hospital laboratories	585	217 (37.1)	100 (46.1)
University and/or research	1164	390 (33.6)	146 (37.4)
Analysis under dept. of pathology (*n* = 2161)^b^	2161	769 (35.6)	320 (41.6)
Yes	1869	651 (34.8)	264 (40.6)
No	292	118 (40.4)	56 (47.5)
Accreditation (*n* = 1874)^c^	1874	702 (37.5)*	285 (40.6)*
Accredited	861	306 (35.5)	139 (45.4)
Not accredited	1013	396 (39.1)	146 (36.9)
Part of the analysis outsourced? (*n* = 340)^d^	340	135 (13.2)*	57 (42.2)
Yes	45	24 (53.3)	10 (41.7)
No	295	111 (37.6)	47 (42.3)
Nr of staff (*n* = 2050)^e^	2050	733 (35.8)	305 (41.6)
1–5	1002	353 (35.2)	146 (41.4)
6–10	638	234 (36.7)	83 (35.5)
11–20	284	101 (35.6)	58 (57.4)
> 20	126	45 (35.7)	18 (40.0)
Number of *EGFR* samples tested last year? (*n* = 209)^e^	209	80 (38.3)	32 (40.0)
No clinical samples tested	8	5 (62.5)	2 (40.0)
< 10	2	1 (50.0)	1 (100.0)
10–99	43	16 (37.2)	4 (25.0)
100–249	65	29 (44.6)	13 (44.8)
250–499	65	20 (30.8)	10 (50.0)
> 500	26	9 (34.6)	2 (22.2)
Number of *ROS1* samples tested last year? (*n* = 601)^e^	601	256 (42.6)*	118 (46.1)
No clinical samples tested	32	18 (56.3)	6 (33.3)
< 10	37	18 (48.6)	8 (44.4)
10–99	197	83 (42.1)	40 (48.2)
100–249	128	54 (42.2)	27 (50)
250–499	126	52 (41.3)	21 (40.4)
> 500	81	31 (38.3)	16 (51.6)
Number of *ALK* samples tested last year? (*n* = 1193)^e^	1193	450 (37.7)	188 (41.8)
No clinical samples tested	23	9 (39.1)	4 (44.4)
< 10	25	9 (36.0)	1 (11.1)
10–99	333	118 (35.4)	47 (39.8)
100–249	374	142 (37.9)	66 (46.5)
250–499	281	113 (40.2)	41 (36.3)
> 500	157	59 (37.6)	29 (49.2)
Number of *PD-L1* samples tested last year? (*n* = 491)^e^	491	258 (52.5)	102 (39.5)
No clinical samples tested	23	9 (39.1)	3 (33.3)
< 10	29	18 (62.1)	8 (44.4)
10–99	193	96 (49.7)	33 (34.4)
100–249	117	66 (56.4)	32 (48.5)
250–499	90	47 (52.2)	16 (34.0)
> 500	39	22 (56.4)	10 (45.5)
Number of *KRAS* samples tested last year? (*n* = 221)^e^	221	92 (41.6)*	39 (42.4)
No clinical samples tested	6	5 (83.3)	2 (40.0)
< 10	5	3 (60.0)	0 (0.0)
10–99	43	21 (48.8)	9 (42.9)
100–249	91	34 (37.4)	13 (38.2)
250–499	53	22 (41.5)	12 (54.5)
> 500	23	7 (30.4)	3 (42.9)
Number of *NRAS* samples tested last year? (*n* = 219)^e^	219	90 (41.1)*	39 (43.3)
No clinical samples tested	7	6 (85.7)	2 (33.3)
< 10	4	2 (50.0)	0 (0.0)
10–99	60	31 (51.7)	16 (51.6)
100–249	90	31 (34.4)	10 (32.3)
250–499	44	14 (31.8)	8 (57.1)
> 500	14	6 (42.9)	3 (50.0)
Number of *BRAF* samples tested last year? (*n* = 207)^e^	207	84 (40.6)	37 (44.0)
No clinical samples tested	10	8 (80.0)	5 (62.5)
< 10	18	4 (22.2)	1 (25.0)
10–99	73	30 (41.1)	15 (50.0)
100–249	65	26 (40.0)	8 (30.8)
250–499	31	12 (38.7)	6 (50.0)
> 500	10	4 (40.0)	2 (50.0)

*p < 0.05. Exact *p* values and odds ratios are shown in Supplemental Data 2A

^a^“Industry” laboratories are those developing diagnostic commercial kits. (Private) laboratories are not within a hospital’s infrastructure. Hospital laboratories included laboratories in private and public hospitals. University and research included education and research hospitals, university hospitals, university laboratories, and anti-cancer centres [14]

^b^Laboratories under the department of pathology are those performing pathology review and the analytical tests in the same department

^c^Accreditation is defined as compliant to ISO 15189 [15] or relevant national standards, such as CAP 15189 [16]

^d^Laboratories who outsourced the analysis sent the samples to another laboratory for any part of the TTP, being either neoplastic cell percentage estimation, DNA extraction, or the genomic analysis

^e^The number of staff involved in the TTP and annual samples tested were used a measure of the size and experience of the laboratory

*ALK*, ALK receptor tyrosine kinase; *BRAF*, B-Raf proto-oncogene; *EGFR*, epidermal growth factor receptor; *EQA*, external quality assessment; *KRAS*, KRAS proto-oncogene; *N*, number; *NRAS*, NRAS proto-oncogene; *PD-L1*, programmed death ligand 1; *ROS1*, ROS proto-oncogene 1; *TTP*, total test process

Laboratories accredited conform ISO 15189 were less likely to receive the survey, as well as laboratories testing a larger number of annual samples for *ROS1*, *KRAS*, or *NRAS*, but not for the other markers. On the contrary, laboratories (*n* = 45) who outsourced a part of their analysis were more probable to receive the survey. Exact *p* values and corresponding odds ratios (ORs) are shown in Supplemental Data 2A. Of the 45 respondents mentioning that they outsourced a part of the analysis, 15 outsourced the variant analysis itself, 6 outsourced both the DNA extraction and variant analysis, and 24 sent the samples to another laboratory for pathology review. There was no difference in the chance to receive the survey based on the laboratory’s setting (university or community hospital), or number of personnel (Table 2).

Of the 791 surveys that were sent, 325 (39.8%) responses were received by 185 unique laboratories (58.4%) from 34 countries (Table 1). On average, the responses were received within 22.5 days (min. 1, max. 211, median 15 days). 139/325 (42.8%) responses were received within the first 2 weeks (no reminder sent), 116 (35.7%) after the first reminder, and 70 (21.5%) after two reminders. The response time or number of reminders sent was not related to the laboratory characteristics (Supplemental Data 2A).

Accredited laboratories were more likely to return the completed survey compared to not accredited laboratories. Other factors did not influence the likelihood of responding to the received survey (Table 2).

### Time point in the total test process and cause of deviating EQA results

Of the 988 NSCLC and 179 mCRC cases with a deviating EQA result between 2015 and 2018, data was obtained for 424 (42.9%) NSCLC and 90 (50.3%) mCRC cases.

For the NSCLC EQA schemes (*n* = 424), errors occurred mostly in the post-analytical (48.1%) phase (Table 3). For the digital cases, the majority of problems occurred in the post-analytical phase, given that these cases only comprised interpretation of pre-made images. This with the exception of some laboratories who implied a problem during the pre-analytical or analytical phase, when the images were created. For analysis of the FFPE samples, mainly post-analytical errors were observed for FISH and IHC, except for *ALK* FISH with 44.0% (*n* = 25) analytical issues. During the IHC technical assessment, the staining quality of the applied protocol was evaluated, which is reflected in a high percentage of analytical issues as contributing factors for problems. For variant analysis, causes were mostly post-analytical for *EGFR* testing (47.2%, *n* = 108) but analytical for *KRAS* (53.8%, *n* = 13) and *BRAF* (100.0%, *n* = 3) testing. In the mCRC EQA schemes, all cases were tested by variant analysis, and results (*n* = 90) revealed mainly issues during the analytical phase itself (42.2%), but percentages varied depending on the marker of analysis.

**Table 3:** Time point of deviating EQA results in the different subschemes

				Error phase (%)			
			*N* (total = 514)	Pre-analytical	Analytical	Post-analytical	Unknown
**NSCLC**	**Technique**	**Marker**	**424**	**15.3**	**30.2**	***48.1***	**6.4**
Digital cases	FISH (interpretation only)	*ALK*	7	0.0	0.0	*100.0*	0.0
		*ROS1*	33	3.0	3.0	*87.9*	6.1
	IHC (interpretation only)	PD-L1	30	6.7	3.3	*83.3*	6.7
FFPE samples	FISH	*ALK*	25	*44.0*	20.0	28.0	8.0
		*ROS1*	49	30.6	18.4	*36.7*	14.3
	IHC	ALK	31	16.1	38.7	*41.9*	3.2
		ROS1	17	5.9	35.3	*52.9*	5.9
		PD-L1	63	19.0	12.7	*55.6*	12.7
	IHC (technical assessment)	ALK	36	13.9	*80.6*	5.6	0.0
		ROS1	6	16.7	*50.0*	33.3	0.0
		PD-L1	3	*33.3*	*33.3*	0.0	*33.3*
	Variant analysis	*EGFR*	108	10.2	39.8	*47.2*	2.8
		*KRAS*	13	0.0	*53.8*	46.2	0.0
		*BRAF*	3	0.0	*100.0*	0.0	0.0
**mCRC**	**Technique**	**Marker**	**90**	**30.0**	***42.2***	**24.4**	**3.3**
FFPE samples	Variant analysis	*KRAS*	53	28.3	*47.2*	18.9	5.7
		*NRAS*	12	25.0	33.3	*41.7*	0.0
		*BRAF*	10	30.0	30.0	*40.0*	0.0
		WT	10	40.0	*50.0*	10.0	0.0
		No neoplastic cells	5	*40.0*	20.0	*40.0*	0.0

For *ALK* and *ROS1* analysis, participation to the FISH subschemes automatically enrolled the laboratory for interpretation of digital FISH cases besides the FFPE cases [12]. Expression of programmed death ligand 1 (*PD-L1*) was assessed (since 2017) by providing FFPE samples for IHC and digital cases for interpretation of the IHC stain. For mCRC EQA schemes, one of the provided colon cases included a case without neoplastic cells to verify the testing practice of the participants in this case. The most frequent reported time points of occurrence are italicized. Start and endpoints of phases in this study were defined based on definitions in ISO 15189 (clauses 3.14 and 3.15) [15]. The pre-analytical phase was communicated in the survey as the time from sample reception until selection and estimation of the neoplastic cell percentage during pathologist review (for variant analysis) and until sample pre-treatment (for FISH or IHC). The analytical phase started from DNA extraction (if applicable) and the actual biomarker test, i.e., all steps of mutation analysis, gene rearrangement, or IHC analysis according to the pre-determined protocol. The post-analytical phase occurred between the readout of the analytical results (interpretation of mutation analysis curves, of the staining intensity/pattern, or reading of the split/single FISH nuclei), and reporting of the results, in this case when entering the results in the electronic EQA datasheets. *ALK*, ALK receptor tyrosine kinase; *BRAF*, B-Raf proto-oncogene; *EGFR*, epidermal growth factor receptor; *EQA*, external quality assessment; *FFPE*, formalin-fixed paraffin embedded; *FISH*, fluorescence in situ hybridization; *IHC*, immunohistochemistry; *KRAS*, KRAS proto-oncogene; *mCRC*, metastatic colorectal cancer; *N*, number; *NRAS*, NRAS proto-oncogene; *NSCLC*, non-small cell lung cancer; *PD-L1*, programmed death ligand 1; *ROS1*, ROS proto-oncogene 1; *WT*, wild-type

Analyzing the underlying causes (Table 4), both interpretation of the digital cases and IHC of the FFPE samples were prone to interpretation errors. For FISH analysis of FFPE cases, problems with the provided EQA material were most often reported. During the technical assessment, problems with the reagents were detected for ALK IHC, versus methodological problems for ROS1 IHC. For PD-L1, reasons of suboptimal staining quality were dispersed. For variant analysis in NSCLC, methodological issues were the main sources of errors, while for variant analysis in mCRC, the underlying causes varied also depending on the analyzed marker.

**Table 4:** Error causes behind deviating EQA results in the different subschemes

				Error cause (%)							
			*N* (total = 514)	Interpretation error	Methodological problem	Problem with EQA material	Reagent problem	Clerical error	Personnel error	Technical/equipment	Unknown/other
**NSCLC**	**Technique**	**Marker**	**424**	***31.8***	**18.2**	**13.0**	**10.4**	**8.7**	**5.0**	**4.7**	**8.3**
Digital cases	FISH (interpretation only)	*ALK*	7	*71.4*	0.0	0.0	0.0	14.3	14.3	0.0	0.0
		*ROS1*	33	*66.7*	0.0	6.1	3.0	3.0	9.1	0.0	12.1
	IHC (interpretation only)	PD-L1	30	*76.7*	0.0	10.0	0.0	0.0	0.0	0.0	13.3
FFPE samples	FISH	*ALK*	25	16.0	4.0	*32.0*	12.0	12.0	4.0	12.0	8.0
		*ROS1*	49	26.5	2.0	*34.7*	10.2	0.0	8.2	4.1	14.3
	IHC	ALK	31	*25.8*	16.1	3.2	*25.8*	12.9	0.0	12.9	3.2
		ROS1	17	*29.4*	17.6	5.9	17.6	23.5	0.0	0.0	5.9
		PD-L1	63	*39.7*	3.2	17.5	7.9	11.1	6.3	1.6	12.7
	IHC (technical assessment)	ALK	36	0.0	22.2	2.8	*44.4*	0.0	5.6	25.0	0.0
		ROS1	6	16.7	*33.3*	16.7	16.7	16.7	0.0	0.0	0.0
		PD-L1	3	0.0	0.0	*33.3*	*33.3*	0.0	0.0	0.0	*33.3*
	Variant analysis	*EGFR*	108	25.0	*41.7*	8.3	0.9	11.1	5.6	0.9	6.5
		*KRAS*	13	15.4	*53.8*	0.0	0.0	30.8	0.0	0.0	0.0
		*BRAF*	3	0.0	*100.0*	0.0	0.0	0.0	0.0	0.0	0.0
**mCRC**	**Technique**	**Marker**	**90**	**10.0**	***31.1***	**13.3**	**8.9**	**10.0**	**16.7**	**5.6**	**4.4**
FFPE samples	Variant analysis	*KRAS*	53	0.0	*37.7*	9.4	7.5	11.3	20.8	7.5	5.7
		*NRAS*	12	*25.0*	8.3	8.3	*25.0*	16.7	16.7	0.0	0.0
		*BRAF*	10	*30.0*	*30.0*	20.0	0.0	10.0	10.0	0.0	0.0
		WT	10	10.0	*40.0*	*40.0*	0.0	0.0	0.0	10.0	0.0
		No neoplastic cells	5	*40.0*	0.0	0.0	20.0	0.0	20.0	0.0	20.0

For *ALK* and *ROS1* analysis, participation to the FISH subschemes automatically enrolled the laboratory for interpretation of digital FISH cases besides the FFPE cases [12]. Expression of programmed death ligand 1 (*PD-L1*) was assessed (since 2017) by providing FFPE samples for IHC and digital cases for interpretation of the IHC stain. For mCRC EQA schemes, one of the provided colon cases included a case without neoplastic cells to verify the testing practice of the participants in this case. The most frequent reported causes are italicized. A more detailed description of the definitions for the different error causes is given in Supplemental Data 3

*ALK*, ALK receptor tyrosine kinase; *BRAF*, B-Raf proto-oncogene; *EGFR*, epidermal growth factor receptor; *EQA*, external quality assessment; *FFPE*, formalin-fixed paraffin embedded; *FISH*, fluorescence in situ hybridization; *IHC*, immunohistochemistry; *KRAS*, KRAS proto-oncogene; *mCRC*, metastatic colorectal cancer; *N*, number; *NRAS*, NRAS proto-oncogene; *NSCLC*, non-small cell lung cancer; *PD-L1*, programmed death ligand 1; *ROS1*, ROS proto-oncogene 1; *WT*, wild-type

The time point in the TTP and cause of the problems differed significantly between the indication (NSCLC vs. mCRC), markers tested and techniques used (Supplemental Data 2B).

Definitions for the different categories and a more detailed cause of problems are given in Supplemental Data 3. Of all interpretation issues, 135 of 144 were reported in the NSCLC schemes. Of these, 51 (37.8%) were reported during interpretation of the IHC staining intensity, 40 (29.6%) during counting of the positive FISH signals, and to a lesser extent (18.5%) due to an incorrect analysis of PCR curves during variant analysis. Causes for methodological problems reported in both schemes (*n* = 105) occurred mostly because the laboratories were unaware that the variant tested in the scheme was not included in their analysis method (35.2%) or the method had an insufficient sensitivity to detect the variant at its respective frequency (20.0%).

### Error causes for the different laboratory characteristics

The probability to encounter a specific error cause in one of the phases of the TTP related to the laboratory characteristics as collected in the EQA datasheets is given in Table 5.

**Table 5:** Error phase and cause related to laboratory characteristics and EQA scheme performance

	Error phase			Error cause						
	Pre-analytical	Analytical	Post-analytical	Clerical error	Equipment/technical problem	Interpretation error	Methodological problem	Personnel error	Problem with EQA material	Reagent problem
Laboratory characteristics										
Setting	↔	↔	↔	↔	↔	↔	↔	↔	↔	↔
Accreditation	↔	↔	↔	↔	↔	↔	↔	↔	↔	↓**
Higher nr. of staff involved in biomarker test	↔	↔	↔	↔	↔	↔	↓**	↔	↔	↔
Analysis under dept. of pathology	↓**	↔	↔	↔	↔	↔	↔	↔	↓***	↑*
Higher nr. of samples tested per year	↔	↔	↔	↔	↔	↔	↔	↑*	↔	↔
Change in test methodology in last 12 months	↔	↔	↔	↔	↔	↔	↑*	↔	↔	↔
Methodology type	↔	↔	↔	↔	↔	↔	↔	↔	↔	↔
EQA performance										
Laboratories who detected the error after release of EQA results	↓*	↓**	↑***	↑*	↓**	↑***	↓*	↔	↓***	↔
Laboratories with a higher performance score	↔	↓***	↑***	↔	↓**	↑***	↔	↓**	↑***	↓***
Laboratories who were successful	↔	↔	↔	↔	↔	↔	↔	↔	↔	↔
Laboratories who obtained fewer genotyping errors	↔	↔	↔	↔	↔	↔	↔	↓***	↑***	↔
Laboratories who obtained fewer analysis failures	↓**	↔	↑*	↔	↔	↑*	↔	↔	↓**	↔

↓ Statistical decrease in error phase/cause, ↑ statistical increase in error phase/cause, ↔ no statistical effect observed. *p < 0.05, **p < 0.01, ***p < 0.001. Detailed *p* values and odds ratios are given in Supplemental Data 2C

Start and endpoints of phases in this study were defined based on definitions in ISO 15189 (clauses 3.14 and 3.15) [15]. The pre-analytical phase was communicated in the survey as the time from sample reception until selection and estimation of the neoplastic cell percentage during pathologist review (for variant analysis) and until sample pre-treatment (for FISH or IHC). The analytical phase started from DNA extraction (if applicable) and the actual biomarker test, i.e., all steps of mutation analysis, gene rearrangement, or IHC analysis according to the pre-determined protocol. The post-analytical phase occurred between the readout of the analytical results (interpretation of mutation analysis curves, of the staining intensity/pattern, or reading of the split/single FISH nuclei), and reporting of the results, in this case when entering the results in the electronic EQA datasheets. Laboratories under the department of pathology are those performing pathology review and the analytical tests in the same department. Accreditation is defined as compliant to ISO 15189 [15] or relevant national standards, such as CAP 15189 [16]. The number of staff involved and annual samples tested were used a measure of the size and experience of the laboratory [24]. A change in method represents laboratories who changed their analysis method or protocol in the last 12 months prior to the survey. A more detailed description of the definitions for the different error causes is given in Supplemental Data 3. *EQA*, external quality assessment; *nr.*, number

Pathology laboratories were significantly less probable of making a mistake in the pre-analytical phase and to denote the received sample material as the cause. On the other hand, they more frequently reported reagent problems. Accredited laboratories less frequently encountered reagent problems.

Laboratories with a larger staff number (usually larger laboratories) had a reduced probability of encountering method-related problems. Testing more samples annually increased the chance of a personnel error to occur. Respondents who changed their testing method in the last 12 months prior to the survey were significantly more likely to obtain a problem with that methodology compared to laboratories who did not change anything to their methodology in this period. There was no significant relationship between any of the other causes and the laboratory characteristics (Supplemental Data 2C).

### Detection of errors during the EQA scheme

Post-analytical problems were more likely to be detected after release of the EQA results especially for clerical and interpretation errors (Table 5). On the other hand, pre-analytical and analytical issues, such as equipment/technical or methodological problems and issues with the EQA material, were more likely to be picked up in advance (Table 5).

Laboratories with an error in the pre-analytical phase were more likely to encounter an analysis failure in the scheme. Laboratories with analytical problems more often obtained lower performance scores, and those with post-analytical problems had a significantly higher score, due to the occurrence of fewer technical failures. More specifically, personnel errors, equipment, and reagent problems lowered the score in the EQA scheme, while laboratories reporting a problem with the material were more likely to obtain a technical failure. Exact *p* values and ORs are shown in Supplemental Data 2C.

The EQA participants undertook specific corrective actions, which were significantly linked to the time in the TPP and cause (Supplemental Fig. 1). Respondents with a personnel error more often had an analysis error in the subsequent EQA scheme, but there was no effect by any other error cause on the performance criteria in the next EQA scheme [24].

## Discussion

Several studies have evaluated the longitudinal improvement of biomarker testing in NSCLC and mCRC for different laboratories, samples, and methods [4–13]. Even though error rates are published [4–13] and some providers request root cause analyses, no information is yet available on the underlying causes for deviating EQA results in the laboratories for molecular oncology.

### Response to root cause surveys

Our data on root causes of deviating EQA results demonstrated that laboratories who are accredited or test more samples annually (for *ROS1*, *KRAS* and *NRAS*) were less likely to receive the survey. Keeping in mind that the surveys were sent only to participants with deviating results, these findings are not surprising. It has been described that accredited laboratories testing more samples demonstrated a better performance in the EQA schemes [14]. In contrast, laboratories that outsourced (a part of) their analysis reported more EQA errors. ISO 15189 states that the laboratory shall have a documented procedure for selecting and evaluating referral laboratories, and is responsible for monitoring their quality [15]. More investigations are needed on which elements of the TTP are being outsourced in routine, the structure of laboratory networks, and how high quality is ensured.

Accredited laboratories were also more likely to reply to the survey. Participation to quality improvement projects such as survey completion or workshop attendance [25] has previously shown to increase EQA performance in mCRC and might contribute to the better performance for accredited participants. We acknowledge that not all countries have responded, and error causes might shift when taking into account data from non-respondents. Nevertheless, with data from 185 laboratories worldwide which encompassed 44.0% of the incorrect samples, this is a valuable first assessment of causes underlying deviating EQA results. The uniform taxonomy and participant-tailored surveys allowed to compare the results between the different survey rounds. A continued follow-up might be useful to evaluate if the conclusions are still valid when evaluating more respondents, as well as for other predictive markers currently not included in the schemes.

### Time point in the total test process and cause of deviating EQA results

The causes of deviating EQA outcomes were related to the indication (NSCLC or mCRC) and included subschemes. It must be noted that for the FFPE samples, more interpretation problems were reported for *ROS1* compared to *ALK*, even when tested by the same technique type (FISH or IHC) and even more so for PD-L1 IHC (Table 4). This is consistent with previously reported increased error rates for *ROS1* compared to *ALK*, explained by an increased experience with *ALK*, as *ROS1* testing was only approved since 2016 [12]. In the survey period, fewer guidelines were thus available for *ROS1* interpretation, and no Food and Drug Administration-approved companion diagnostic (which was the case for *ALK*). For *PD-L1*, a similar assumption can be made as it is only recently required for testing and its interpretation poses additional challenges due the availability of different commercially antibodies with varying cut-offs for positivity for different therapies [26].

In case sample problems were reported for FISH, the most prominent reasons were suboptimal sample quality (20.9%) or too few neoplastic cells (14.9%) (Supplemental Data 3). Estimation of the neoplastic cell content in EQA schemes has been reported as highly variable [27]. Nevertheless, materials were carefully validated beforehand to have sufficient neoplastic cells and lacking tumor heterogeneity, and other peers were able to successfully analyze them. Even though digital FISH cases only assess the post-analytical phase, for two cases, the survey respondents mentioned a problem during creation of the images at the pre- or analytical phase to be at the basis of the interpretation error (Table 3).

For variant analysis, the laboratories frequently reported the lack of a specific variant in the analysis method (Supplemental Data 3), especially for mCRC (17.8%) compared to NSCLC (5.0%). This is a well-known problem, as in 2013, the drug label for cetuximab and panitumumab was extended to include codons 12, 13, 59, 61, 117, and 146 for both the *KRAS* and *NRAS* genes, but not all laboratories have adapted their testing strategy [11]. Also, insufficient method sensitivity was reported, as well as misinterpretation of obtained sequencing curves (e.g., results around the threshold), which are especially important in routine for variants at low frequencies such as *EGFR* c.2369C>T p.(Thr790Met) LRG_304p1. The number of errors reported in wild-type cases was too low to make solid assumptions.

The specific causes suggest that EQA providers could benefit from requesting root cause analyses after the schemes to provide more tailored education to participants. For instance, the provision of digital or paper-based cases to assess interpretation or variant classifications could aid in the interpretation for specific markers. Given the broad variety of methodologies used by the participants completing the survey, the performance of these methods might have further contributed to the error causes. Indeed, different performances have been reported depending on the applied PD-L1 IHC clones (personal observations), ALK IHC clones, or *EGFR* variant analysis method in the same ESP NSCLC EQA schemes, and depending on *RAS* analysis methods in the ESP mCRC EQA schemes [11, 13, 28]. Challenging samples might be included (albeit educational) with rare variants to assess the inclusion of all relevant mutations or their detection at low allele frequencies. Schemes should thus be fit for purpose [19] and should cover the entire examination process as required by ISO 15189 [15]. As the samples in the EQA scheme were pre-cut and labeled, several pre-analytical steps were outside the study scope. Research on routine cases is advised to assess problems during sample embedding, cutting, or labelling.

### Error causes for the different laboratory characteristics

Previous longitudinal results indicated that experience (by accreditation, a research setting, or testing more annual samples) positively affected EQA scores [14]. Our findings revealed that personnel errors increased when testing more samples, probably due to the increased work pressure. Laboratory automation might be the way forward to reduce these errors. Also, laboratories with an increased number of staff had fewer method-based errors, by the probable larger capacity of professionally trained personnel to perform a certain method [29]. Accredited laboratories less frequently had a reagent problem, possibly due to working according to standard operating procedures. As these reagent problems significantly lowered the EQA performance, this might explain their previously better performance.

Our data also revealed that laboratories operating under the department of pathology less often reported sample-related issues (Table 3), but more frequently encountered reagent problems, as they were more frequently involved in IHC analysis compared to molecular laboratories. The positive influence of pathology review in decreasing specimen problems in this study stresses its importance to obtain accurate results further downstream the TTP.

We did not observe a difference in error rates concerning the method type (i.e., NGS versus pyrosequencing), in agreement with previous studies [14]. However, we observed that a change in test method during the last year resulted in significantly more method-related error causes, highlighting the importance of test validation before implementation in clinical practice.

### Detection of errors during the EQA scheme

Post-analytical clerical and interpretation problems were less likely detected before release of the results (Table 5) in contrast to equipment, methodological, and sample-related problems. This seems logical, given that post-analytical issues occur closer to reporting of the results and have less time to be picked up by a quality control step. This might explain the higher error rates previously reported for *ROS1* compared to *ALK* [12], as this marker now indeed revealed a large fraction of clerical and interpretation causes.

Looking at the current scheme performance (Table 5), errors in the pre-analytical phase were more prominent for participants with lower performance scores and more technical failures. This again underlines the importance of pre-analytic quality control to prevent technical failures resulting from selecting insufficient neoplastic cells [27].

None of the causes had a significant effect on future scheme performances except for personnel errors. In this case, laboratories most frequently responded by retraining their staff [24]. Also, for the majority of errors, an appropriate corrective action was undertaken (Supplemental figure 1).

## Conclusions

To conclude, causes of deviating EQA results were indication, marker, and technique dependent. The phase and underlying cause differently affected the EQA performance, either by an increase in test failures or false-positive/false-negative results. Our findings advocate using surveys by EQA providers to specifically tailor the schemes for set-up, feedback, and offered sample types. Timely quality checks aid to uncover deviating results and should be additionally implemented in the post-analytical phase as these errors were often not identified in the laboratory. Accredited laboratories were more likely to respond and had fewer reagent problems, which could explain their previously reported better performance. We detected an important effect of pathology review to reduce technical failures and of protocol changes to increase method-related problems.

## Supplementary information

ESM 1 (PDF 403 kb)

ESM 2 (PDF 378 kb)

ESM 3 (PDF 444 kb)

ESM 4 (PDF 339 kb)

## Data Availability

The datasets generated during and/or analyzed during the current study are available from the corresponding author on reasonable request.

## Abbreviations

*ALK*: ALK receptor tyrosine kinase
*BRAF*: B-Raf proto-oncogene
CAPA: Corrective/preventive action
CI: Confidence interval
*EGFR*: Epidermal growth factor receptor
EQA: External quality assessment
ESP: European Society of Pathology
FFPE: Formalin-fixed paraffin embedded
FISH: Fluorescence in situ hybridization
GEE: Generalized estimating equations
IHC: Immunohistochemistry
ISO: International Organization for Standardisation
*KRAS*: KRAS proto-oncogene
mCRC: Metastatic colorectal carcinoma
NGS: Next-generation sequencing
*NRAS*: NRAS proto-oncogene
NSCLC: Non-small-cell lung cancer
OR: Odds ratio
*PD-L1*: Programmed death ligand 1
*ROS1*: ROS proto-oncogene 1
TPS: Tumor proportion score
TTP: Total test process
WT: Wild-type
