# Supplementary material for: Causes behind error rates for predictive biomarker testing: the utility of sending post-EQA surveys
Source: Virchows Arch. 2020 Nov 23;478(5):995–1006. doi: 10.1007/s00428-020-02966-7 (PMC8099794; doi:10.1007/s00428-020-02966-7)
Supplement: Supplementary file 1 — (PDF 403 kb) [file 428_2020_2966_MOESM1_ESM.pdf]

Title	Causes behind error rates for predictive biomarker testing: the utility of sending post-EQA surveys.
Journal	Virchows Archiv
Authors	Keppens Cleo, Schuuring Ed, Dequeker MC Elisabeth
Correspondence	Prof. Dr. Elisabeth Dequeker, University of Leuven,<br>Department of Public Health and Primary Care,<br>Biomedical Quality Assurance Research Unit,<br>Kapucijnenvoer 35d, Box 7001, Leuven 3000, Belgium.<br>Tel: +3216 345881, E-mail: els.dequeker@kuleuven.be
File	**Supplemental Data 1: Overview of the survey questions**

Information on the used methodology for sample analysis, sample outcomes and laboratory performance are standardly analyzed at the end of an EQA scheme. These questions include extra information collected to perform additional research. EQA scheme.

- ○ One answer possible
- ☐ Multiple answers possible

Abbreviations: *ALK*, ALK receptor tyrosine kinase; EQA, external quality assessment; FISH, fluorescence in-situ hybdrisation; SOP, standard operating procedure; Q, question.

I. Questions included in the EQA datasheet during the time of results submission

Q1: Settings for your laboratory:

- O Community Hospital
- O University Hospital
- O University
- O Private
- O Industry
- O Private Hospital
- O Other (Please specify)

Q2: Is the analysis performed under the authority of the department of pathology?

- O Yes
- O No

Q3: Is part of this analysis performed by another laboratory?

- O No
- O Yes

Q3.1: If yes, which parts?

Q4: How many people in your laboratory are involved in oncology biomarker testing (full time equivalent). Include dedicated administrative and technical support.

- O 1-5
- O 6-10
- O 11-15
- O 15-20
- O >20

Q5: Is your laboratory accredited or certified?

- O Yes
- O No

Q5.1: If yes, according to which standard?
Q5.2: If yes, by which organization?

Q6: How many (approximately) biopsies were tested for this marker by your laboratory in the last 12 months (for diagnostics purposes only, not for research)?

- O 0
- O 1-10
- O 10-49
- O 50-99
- O 100-249
- O 250-499
- O 500-999
- O >1000

II. Additional survey questions sent at the end of the EQA scheme

INTRODUCTION: Please find below a short questionnaire on the follow-up the EQA scheme results. You can select the appropriate option in the dropdown menu, which will become visible when clicking on 'please choose'. If required, please specify your answer in more detail in the column next to the selected option. Your contribution to this study is highly appreciated.

EQA ID: *Prefilled with participants anonymous number to the EQA scheme e.g. 2018LUNG0001*

A. CASE-SPECIFIC QUESTIONS *Repeated for all cases in which an error occurred*

SUBSCHEME: *Prefilled with relevant marker and subscheme e.g. ALKFISH*

CASE NR: *Prefilled with the sample label and error type e.g. L18.ALKFISH1: false-negative*

Q1: During which phase in the test process did the error occur?

- O Pre-analytical phase
- O Analytical phase
- O Post-analytical phase
- O I don't know

Q2: What was the cause of this error?

- O Clerical error
- O Methodological problem
- O Equipment problem
- O Technical problem
- O Reagent problem
- O Personnel error
- O Interpretation error
- O Problem with EQA material
- O I don't know because not documented
- O I don't know although documented
- O Other (please specify)

Q3: Please shortly specify the cause of the error:

Q4: Which corrective/preventive action was taken for this error?

- O Implement/optimize documentation (procedure)
- O Protocol revision (technical)
- O Change method/control tissue
- O Contact company
- O Staff training (internal)
- O Staff training (external)
- O None
- O I don't know
- O Other (please specify)

Q4.1: If staff training, please clarify which kind:
e.g. Additional EQA participation, workshop, internal SOP revision,...

Q5: Was this error detected before or after the EQA results were released?

- O Before
- O After

Q5.1: If before, please specify how the error was identified:

B. GENERAL QUESTIONS *To be completed only once*

Q1: Did you change anything to the test protocol/method in the last 12 months?

- O Yes
- O No

Q1.1: If yes, please specify what was changed:

THANK YOU FOR YOUR PARTICIPATION.
