# Supplementary material for: Causes behind error rates for predictive biomarker testing: the utility of sending post-EQA surveys
Source: Virchows Arch. 2020 Nov 23;478(5):995–1006. doi: 10.1007/s00428-020-02966-7 (PMC8099794; doi:10.1007/s00428-020-02966-7)
Supplement: Supplementary file 2 — (PDF 378 kb) [file 428_2020_2966_MOESM2_ESM.pdf]

Title	Causes behind error rates for predictive biomarker testing: the utility of sending post-EQA surveys.
Journal	Virchows Archiv
Authors	Keppens Cleo, Schuuring Ed, Dequeker MC Elisabeth
Correspondence	Prof. Dr. Elisabeth Dequeker, University of Leuven,<br>Department of Public Health and Primary Care,<br>Biomedical Quality Assurance Research Unit,<br>Kapucijnenvoer 35d, Box 7001, Leuven 3000, Belgium.<br>Tel: +3216 345881, E-mail: els.dequeker@kuleuven.be
File	**Supplemental Data 2: Exact p-values and odds ratios for the statistical analyses**.

Logistic regression models with generalized estimating equations (GEE) were used. Binary outcome variables were analyzed by logistic regression models. Ordinal and categorical outcome variables were analyzed by proportional odds models. For categorical variables (laboratory setting, methodology used, scheme marker and scheme technique), pairwise comparisons were only calculated if the global p-value was significant. In case of a significant effect (shown in bold), results are presented as odds ratios (ORs) with 95% confidence intervals [95% CI]. Only characteristics on which information was available is presented. Ratios > (<) 1 reflect a higher (lower) probability for a higher level (+1 category for ordinal variables e.g. for the number of samples tested) or for the first level compared to the second level (for binary variables e.g. accreditation or no accreditation).

A. Exact statistics on survey distribution and response for the different laboratory characteristics

-	Probability of survey receipt	Probability of survey response	Number of reminders sent	Survey response time (days)
Setting (global	p=0.2821	p=0.3776	p=0.1237	p=0.6553
Analysis under dept. of pathology	p=0.1236	p=0.4205	p=0.7483	p=0.2790
Accreditation	**p=0.0382, 0.785 [0.625;0.987]**	**p=0.0290, 1.533 [1.045;2.250]**	p=0.8682	p=0.1564
Part of the analysis otusourced	**p=0.0221, 1.930 [1.099;3.389]**	p=0.6482	p=0.3545	p=0.1070
Nr of staff (ordinal)	p=0.6702	p=0.6934	p=0.8310	p=0.1634
Number of *EGFR* samples tested last year (ordinal)	p=0.1413	p=0.6290	p=0.9015	p=0.6983
Number of *ROS1* samples tested last year (ordinal)	**p=0.0352, 0.875 [0.773;0.991]**	p=0.6403	p=0.5695	p=0.2906
Number of *ALK* samples tested last year (ordinal)	p=0.7205	p=0.6376	p=0.6393	p=0.9355
Number of *PD-L1* samples tested last year (ordinal)	p=0.8463	p=0.2495	p=0.8126	p=0.4428
Number of *KRAS* samples tested last year (ordinal)	**p=0.0345, 0.744 [0.565;0.979]**	p=0.3284	p=0.1765	p=0.1252
Number of *NRAS* samples tested last year (ordinal)	**p=0.0127, 0.672 [0.492;0.919]**	p=0.5141	p=0.2495	p=0.1270
Number of *BRAF* samples tested last year (ordinal)	p=0.5457	p=0.3106	p=0.5684	p=0.3153

B. Error phase and cause related the scheme indication, analyzed marker or technique types

-	Indication (mCRC compared to NSCLC)	Marker (global test)	Per marker comparisons (significant results only)	Technique type (global test)	Per technique comparisons (significant results only)
-	Phase				
Pre-analytical	**p=0.0417, 1.956 [1.026;3.730]**	p=0.7889	-	**p=0.0006**	FISH digital vs. IHC 0.109 [0.013;0.899], p=0.0395<br>FISH vs. FISH digital 28.366 [3.449;233.31], p=0.0020<br>FISH vs. IHC 3.098 [1.380;6.955], p=0.0063<br>FISH vs. IHC digital 9.284 [1.844;46.736], p=0.0070<br>FISH vs. IHC microscopic analysis 4.323 [1.496;12.488], p=0.0070<br>FISH vs. variant analysis 3.716 [1.796;7.689], p=0.0004
Analytical	**p=0.0394, 1.759 [1.028;3.010]**	**p<0.0001**	ALK vs. PD-L1 6.679 [3.081;14.480], p<0.0001<br>ALK vs. ROS1 4.020 [1.969;8.205], p=0.0002<br>BRAF vs. PD-L1 7.206 [1.593;32.604], p=0.0105<br>EGFR vs. PD-L1 4.501 [2.051;9.881], p=0.0002<br>EGFR vs. ROS1 2.709 [1.314;5.586], p=0.0071<br>KRAS vs. PD-L1 8.791 [3.654;21.150], p<0.0001<br>KRAS vs. ROS1 5.290 [2.344;11.943] , p<0.0001<br>PD-L1 vs. WT/without tumor 0.188 [0.071;0.498], p=0.0008<br>ROS1 vs. WT/without tumor 0.313 [0.125;0.783], p=0.0132	**p<0.0001**	FISH digital vs. IHC 0.082 [0.010;0.645], p=0.0177<br>FISH digital vs. IHC microscopic analysis 0.009 [0.001;0.074], p<0.0001<br>FISH digital vs. variant analysis 0.035 [0.005;0.270], p=0.0014<br>FISH vs. FISH digital 10.278 [1.258;83.981], p=0.0298<br>FISH vs. IHC digital 8.354 [1.004;69.542], p=0.0496<br>FISH vs. IHC microscopic analysis 0.090 [0.035;0.234], p<0.0001<br>FISH vs. variant analysis 0.363 [0.181;0.728], p=0.0045<br>IHC digital vs. IHC microscopic analysis 0.011 [0.001;0.092], p<0.0001<br>IHC digital vs. variant analysis 0.043 [0.006;0.337], p=0.0028<br>IHC vs. IHC digital 9.925 [1.243;79.260], p=0.0305<br>IHC vs. IHC microscopic analysis 0.107 [0.045;0.253], p<0.0001<br>IHC vs. microscopic analysis variant analysis 4.029 [1.820;8.916], p=0.0006<br>IHC vs. variant analysis 0.431 [0.242;0.767], p=0.0044

Post-analytical	**p=0.0001, 0.320 [0.179;0.573]**	**p<0.0001**	ALK vs. EGFR 0.446 [0.234;0.850], p=0.0144<br>ALK vs. PD-L1 0.176 [0.089;0.346], p<0.0001<br>ALK vs. ROS1 0.281 [0.145;0.542], p=0.0002<br>BRAF vs. PD-L1 0.191 [0.041;0.883], p=0.0342<br>EGFR vs. KRAS 2.903 [1.333;6.321], p=0.0074<br>EGFR vs. PD-L1 0.394 [0.203;0.766], p=0.0061<br>KRAS vs. PD-L1 0.136 [0.060;0.310], p<.0001<br>KRAS vs. ROS1 0.217 [0.097;0.482], p=0.0002<br>NRAS vs. PD-L1 0.164 [0.028;0.967], p=0.0458<br>PD-L1 vs. WT/without tumor 5.000 [2.036;12.278], p=0.0005<br>ROS1 vs. WT/without tumor 3.133 [1.306;7.519], p=0.0107	**p<0.0001**	FISH digital vs. IHC 14.520 [3.178;66.351], p=0.0006<br>FISH digital vs. IHC microscopic analysis 179.54 [29.701;1085.3], p<0.0001<br>FISH digital vs. variant analysis 29.418 [6.586;131.40], p<0.0001<br>FISH vs. FISH digital 0.032 [0.007;0.149], p<0.0001<br>FISH vs. IHC 0.459 [0.228;0.925], p=0.0295<br>FISH vs. IHC digital 0.063 [0.016;0.246], p<0.0001<br>FISH vs. IHC microscopic analysis 5.674 [1.719;18.729], p=0.0045<br>IHC digital vs. IHC microscopic analysis 90.381 [17.635;463.22], p<0.0001<br>IHC digital vs. variant analysis 14.809 [4.067;53.920], p<0.0001<br>IHC vs. IHC digital 0.137 [0.037;0.509], p=0.0031<br>IHC vs. IHC microscopic analysis 12.365 [3.961;38.598], p<0.0001<br>IHC vs. variant analysis 2.026 [1.167;3.516], p=0.0122<br>IHC microscopic vs. analysis variant analysis 0.164 [0.054;0.499], p=0.0016
Cause					
Clerical error	p=0.9656	p=0.3872	-	p=0.6567	-
Equipment /technical problem	p=0.8436	p=0.2370	-	p=0.0764	-
Interpretation error	**p=0.0003, 0.236 [0.110;0.509]**	**p<0.0001**	ALK vs. PD-L1 0.135 [0.061;0.295], p<.0001<br>ALK vs. PD-L1 0.135 [0.061;0.295], p<0.0001<br>ALK vs. ROS1 0.224 [0.103;0.485], p=0.0002<br>BRAF vs. PD-L1 0.076 [0.008;0.715], p=0.0244<br>EGFR vs. KRAS 5.061 [1.522;16.827], p=0.0083<br>EGFR vs. PD-L1 0.234 [0.112;0.490], p=0.0001<br>EGFR vs. ROS1 0.389 [0.189;0.803], p=0.0109<br>KRAS vs. PD-L1 0.046 [0.014;0.156], p<0.0001<br>KRAS vs. ROS1 0.077 [0.023;0.255], p<0.0001<br>KRAS vs. WT/without tumor 0.173 [0.045;0.664], p=0.0107<br>PD-L1 vs. WT/without tumor 3.741 [1.412;9.913], p=0.0081	**p<0.0001**	FISH digital vs. IHC 6.574 [2.341;18.460], p=0.0004<br>FISH digital vs. IHC microscopic analysis 224.64 [23.192;2175.8], p<0.0001<br>FISH digital vs. variant analysis 17.706 [6.354;49.339], p<0.0001<br>FISH vs. FISH digital 0.082 [0.026;0.256], p<0.0001<br>FISH vs. IHC digital 0.031 [0.007;0.144], p<0.0001<br>FISH vs. IHC microscopic analysis 18.365 [2.177;154.93], p=0.0076<br>IHC digital vs. IHC microscopic analysis 589.38 [48.979;7092.2], p<0.0001<br>IHC digital vs. variant analysis 46.455 [11.064;195.06], p<0.0001<br>IHC vs. IHC digital 0.058 [0.014;0.244], p=0.0001<br>IHC vs. IHC microscopic analysis 34.173 [4.222;276.57], p=0.0010

					IHC vs. variant analysis 2.694 [1.413;5.134], p=0.0027<br>IHC microscopic analysis vs. variant analysis 0.079 [0.010;0.632], p=0.0170
Methodological problem	**p=0.0323, 1.988 [1.060;3.729]**	**p<0.0002**	ALK vs. EGFR 0.178 [0.080;0.393], p<0.0001<br>ALK vs. KRAS 0.146 [0.059;0.362], p<0.0001<br>ALK vs. PD-L1 3.783 [1.136;12.605], p=0.0304<br>BRAF vs. PD-L1 13.881 [2.250;85.640], p=0.0048<br>BRAF vs. ROS1 12.435 [1.991;77.680], p=0.0072<br>EGFR vs. PD-L1 21.311 [6.651;68.284], p<0.0001<br>EGFR vs. ROS1 19.090 [5.891;61.864], p<0.0001<br>KRAS vs. PD-L1 25.833 [7.459;89.468], p<0.0001<br>KRAS vs. ROS1 23.142 [6.672;80.266], p<0.0001<br>PD-L1 vs. WT/without tumor 0.097 [0.025;0.378], p=0.0008<br>ROS1 vs. WT/without tumor 0.108 [0.028;0.423], p=0.0015	p=0.9999	-
Personnel error	**p=0.0157, 3.130 [1.242;7.887]**	p=0.7307	-	p=0.5538	-
Problem with EQA material	p=0.9727	p=0.0984	-	**p<0.0001**	FISH vs. FISH digital 11.922 [2.444;58.169], p=0.0023<br>FISH vs. IHC 4.590 [1.961;10.744], p=0.0005<br>FISH vs. IHC digital 4.861 [1.186;19.912], p=0.0281<br>FISH vs. IHC microscopic analysis 11.520 [2.947;45.031], p=0.0005<br>FISH vs. variant analysis 6.433 [2.968;13.943], p<0.0001
Reagent problem	p=0.6041	**p=0.0019**	ALK vs. EGFR 98.614 [8.839;1100.2], p=0.0002<br>ALK vs. KRAS 14.577 [2.273;93.496], p=0.0049<br>ALK vs. PD-L1 7.526 [2.160;26.229], p=0.0016<br>ALK vs. ROS1 4.102 [1.367;12.310], p=0.0120<br>ALK vs. WT/without tumor 16.153 [1.640;159.07], p=0.0173<br>EGFR vs. PD-L1 0.076 [0.006;0.918], p=0.0426<br>EGFR vs. ROS1 0.042 [0.004;0.466], p=0.0101	**p=0.0003**	FISH digital vs. IHC 0.054 [0.004;0.767], p=0.0312<br>FISH digital vs. IHC microscopic analysis 0.008 [0.000;0.131], p=0.0008<br>FISH vs. IHC microscopic analysis 0.080 [0.017;0.383], p=0.0016<br>FISH vs. variant analysis 11.072 [1.910;64.182], p=0.0075<br>IHC vs. IHC microscopic analysis 0.140 [0.039;0.498], p=0.0025<br>IHC vs. variant analysis 19.273 [3.364;110.41], p=0.0010<br>IHC microscopic analysis vs. variant analysis 137.83 [17.599;1079.4], p<0.0001

C. Error phase and type related to laboratory characteristics and EQA scheme performance

	Phase			Cause						
	Pre-analytical	Analytical	Post-analytical	Clerical error	Equipment/ technical problem	Interpretation error	Methodological problem	Personnel error	Problem with EQA material	Reagent probler
**Laboratory characteristics**										
Setting (global	p=0.9248	p=0.7246	p=0.4205	p=0.8801	p=0.4898	p=0.5094	p=0.5713	p=0.9567	p=0.7651	p=0.2315
Analysis under dept. of pathology	**p=0.0066, 0.358 [0.171;0.749]**	p=0.5150	p=0808	p=0.3203	p=0.0987	p=0.0532	p=0.5349	p=0.6643	**p=0.0003, 0.257 [0.124;0.532]**	**p=0.0471, 8.164 [1.028;64.864]**
Accreditation	p=0.5370	p=0.3152	p=0.1864	p=0.3864	p=0.4136	p=0.0721	p=0.8224	p=0.7102	p=0.7245	**p=0.0057, 0.327 [0.148;0.721]**
Nr of staff (ordinal)	p=0.9125	p=0.2352	p=0.3193	p=0.1067	p=0.6179	p=0.2412	**p=0.0068, 0.604 [0.420;0.869]**	p=0.5495	p=0.4455	p=0.7912
Change in test methodology in last 12 months	p=0.2423	p=0.1403	p=0.6075	p=0.9036	p=0.3940	p=0.6139	**p=0.0104, 2.193 [1.205;3.993]**	p=0.7210	p=0.3317	p=0.2816
Methodology type (global test)	p=0.2477	p=0.7710	p=0.1436	p=0.0634	p=0.6882	p=0.2750	p=0.7494	p=0.6897	p=0.1114	p=0.0826
Higher nr of samples tested (ordinal)	p=0.5644	p=0.9954	p=0.4682	p=0.2861	p=0.4185	p=0.3321	p=0.8427	**p=0.0414, 1.592 [1.019;2.488]**	p=0.6469	0.1682
**EQA performance**										
Error detected after release of EQA results	**p=0.0145, 0.420 [0.210;0.841]**	**p=0.0040, 0.431 [0.244;0.763]**	**p<0.0001, 4.543 [2.326;8.873]**	**p=0.0427, 5.274 [1.057;26.323]**	**p=0.0012, 0.202 [0.077;0.526]**	**p=0.0003, 5.040 [2.128;11.936]**	**p=0.0318, 0.493 [0.259;0.940]**	p=0.1629	**p=0.0009, 0.294 [0.143;0.601]**	p=0.9749
Higher performance score	p=0.5134	**p=0.0004, 0.977 [0.964;0.989]**	**p<0.0001, 1.031 [1.016;1.045]**	p=0.8024	**p=0.0053, 0.969 [0.947;0.990]**	**p<0.0001, 1.051 [1.031;1.070]**	p=0.6905	**p=0.0087, 0.972 [0.951;0.993]**	**p=0.0084, 1.029 [1.007;1.051]**	**p<0.0001, 0.957 [0.940;0.973]**
Successful participation	p=0.2443	p=0.6062	p=0.5808	p=0.1637	p=0.5057	p=0.3045	p=0.8159	p=0.0903	p=0.2655	p=0.9802
Fewer genotyping errors	p=0.3395	p=0.2238	p=0.8378	p=0.1175	p=0.9660	p=0.0609	p=0.2919	**p<0.0001, 0.392 [0.250;0.614]**	**p=0.0001, 2.016 [1.408;2.890]**	p=0.1283
Fewer analysis failures	**p=0.0072, 0.612 [0.429;0.875]**	p=0.7425	**p=0.0192, 1.504 [1.070;1.504]**	p=0.6157	p=0.6614	**p=0.0390, 1.555 [1.022;2.364]**	p=0.5919	p=0.2354	**p=0.0012, 0.561 [0.397;0.795]**	p=0.8858
