# Supplementary material for: Causes behind error rates for predictive biomarker testing: the utility of sending post-EQA surveys
Source: Virchows Arch. 2020 Nov 23;478(5):995–1006. doi: 10.1007/s00428-020-02966-7 (PMC8099794; doi:10.1007/s00428-020-02966-7)
Supplement: Supplementary file 3 — (PDF 444 kb) [file 428_2020_2966_MOESM3_ESM.pdf]

Title	Causes behind error rates for predictive biomarker testing: the utility of sending post-EQA surveys.
Journal	Virchows Archiv
Authors	Keppens Cleo, Schuuring Ed, Dequeker MC Elisabeth
Correspondence	Prof. Dr. Elisabeth Dequeker, University of Leuven,<br>Department of Public Health and Primary Care,<br>Biomedical Quality Assurance Research Unit,<br>Kapucijnenvoer 35d, Box 7001, Leuven 3000, Belgium.<br>Tel: +3216 345881, E-mail: els.dequeker@kuleuven.be
File	Supplemental Data 3: specific error causes reported for the types of problems.

Supplemental data 3: specific error causes reported for the types of problems.

Problems reported	NSCLC (n=424)	mCRC (n=90)	Total (n=514)
**Interpretation error:** Sample processed correctly, but incorrect conclusions are made from the output of the test. E.g. wrong interpretation of the color codes in the FISH probe, as of which the sample is incorrectly denoted as negative. Weak antibody staining incorrectly interpreted as positive without conducting further tests or retesting the sample. Incorrect readout from software output, counting of the same nuclei twice,…	**135 (31.8)**	**9 (10.0)**	**144 (28.0)**
Incorrect interpretation of IHC staining intensity	51 (12.0)	N/A	51 (9.9)
Incorrect interpretation of FISH signals	40 (9.4)	N/A	40 (7.8)
Incorrect interpretation of variant analysis results (e,g, overinterpretation pf PCR curves)	25 (5.9)	7 (7.8)	32 (6.2)
Unspecified	15 (3.5)	0 (0.0)	15 (2.9)
Incorrect interpretation of clinical significance of detected variant (e.g. low frequency variant detected, SNPs)	3 (0.7)	1 (1.1)	4 (0.8)
Unknown	1 (0.2)	1 (1.1)	2 (0.4)
**Methodological problem:** Problems that occur because the method is not optimally suited for all situations presented at the laboratory. E.g. more stringent threshold is required for positivity, more optimal use of antibody concentration is required, a more stringent contamination procedure must be followed, a method is used that did not detect all required markers by regulatory bodies, the method sensitivity is too low	**77 (18.2)**	**28 (31.1)**	**105 (20.4)**
Variant not included in method	21 (5.0)	16 (17.8)	37 (7.2)
Insufficient method sensitivity	18 (4.2)	3 (3.3)	21 (4.1)
Unexpected false-positive/systematic SNP detection	7 (1.7)	3 (3.3)	10 (1.9)
Unknown	6 (1.4)	1 (1.1)	7 (1.4)
Method not suitable for provided material	6 (1.4)	1 (1.1)	7 (1.4)
Software problem methosology	4 (0.9)	3 (3.3)	7 (1.4)
Unexplained weak IHC staining	4 (0.9)	N/A	4 (0.8)
IHC protocol problem	3 (0.7)	N/A	3 (0.6)
Unspecified	3 (0.7)	0 (0.0)	3 (0.6)
Variant analysis reaction failed/cross-reactivity	2 (0.5)	0 (0.0)	2 (0.4)
Weak FISH signals	1 (0.2)	N/A	1 (0.2)
Problem with DNA extraction	0 (0.0)	1 (1.1)	1 (0.2)
Problem during FISH pre-treatment	1 (0.2)	N/A	1 (0.2)
Lack of positive *ALK* control	1 (0.2)	N/A	1 (0.2)
**Problem with EQA material:** Problems that are caused because the EQA material slightly differs from material used for validation of the method, e.g. cell-lines, other thickness of slides,…	**55 (13.0)**	**12 (13.3)**	**67 (13.0)**
Unknown	14 (3.3)	0 (0.0)	14 (2.7)
Suboptimal sample quality	12 (2.8)	2 (2.2)	14 (2.7)
Neoplastic cell percentage	5 (1.2)	5 (5.6)	10 (1.9)
DNA quality	3 (0.7)	5 (5.6)	8 (1.6)
Problem with fixative	6 (1.4)	0 (0.0)	6 (1.2)
Tumor heterogeneity	6 (1.4)	0 (0.0)	6 (1.2)
Sample thickness	5 (1.2)	0 (0.0)	5 (1.0)
Unspecified	3 (0.7)	0 (0.0)	3 (0.6)
Unsuitability for validated procedure	1 (0.2)	0 (0.0)	1 (0.2)

**Problems reported *(continued)***	**NSCLC (n=424)**	**mCRC (n=90)**	**Total (n=514)**
**Reagent problem:** Unexpected problems that occur with reagents for the analysis e.g. background staining, unexplained weak staining, weak probe fluorescence,…	**44 (10.4)**	**8 (8.9)**	**52 (10.1)**
IHC antibody	30 (7.1)	N/A	30 (5.8)
Primers for variant detection	1 (0.2)	8 (8.9)	9 (1.8)
FISH probe	7 (1.7)	N/A	7 (1.4)
excessive IHC background/overstaining	3 (0.7)	N/A	3 (0.6)
Unspecified	2 (0.5)	0 (0.0)	2 (0.4)
Problem during deparaffinization	1 (0.2)	0 (0.0)	1 (0.2)
**Clerical error:** Sample processed correctly, but results were incorrectly entered into the EQA datasheet or the laboratory's own information system/miscommunication between departments.	**37 (8.7)**	**9 (10.0)**	**46 (8.9)**
Mistyping during filling of EQA datasheet	33 (7.8)	9 (10.0)	42 (8.2)
Unspecified	3 (0.7)	0 (0.0)	3 (0.6)
Mistyping during sample re-labelling	1 (0.2)	0 (0.0)	1 (0.2)
**Unknown:** The error is unknown and the cause cannot be traced back because this error was not documented in the past, or the responsible person does not work at the laboratory anymore.	**35 (8.3)**	**3 (3.3)**	**38 (7.4)**
Cause unknown, but problem documented	23 (5.4)	1 (1.1)	24 (4.7)
Cause unknown because problem was not documented	11 (2.6)	2 (2.2)	13 (2.5)
Unspecified	1 (0.2)	0 (0.0)	1 (0.2)
**Personnel error:** Error that occurred due to a mistake of the personnel (besides clerical errors), e.g. not correctly following the manufacturer's instructions, pipetting error, selecting wrong settings on the machine, samples are switched or incorrectly labelled	**21 (5.0)**	**15 (16.7)**	**36 (7.0)**
Samples switched	3 (0.7)	8 (8.9)	11 (2.1)
Error during data analysis (incorrect protocol, plug-inn selected, annotation)	6 (1.4)	4 (4.4)	10 (1.9)
Error during sample labelling	5 (1.2)	0 (0.0)	5 (1.0)
Selection during the neoplastic cell percentage	3 (0.7)	1 (1.1)	4 (0.8)
Unspecified	3 (0.7)	0 (0.0)	3 (0.6)
Error during analysis itself (pipetting error, incorrect protocol selected)	1 (0.2)	2 (2.2)	3 (0.6)
**Technical/equipment problem:** A technical problem occurred with the available hardware/software present in the laboratory. E.g. error message on autostainer, invalid mutation analysis results reported by machine, problems with antibody detection,…	**20 (4.7)**	**5 (5.6)**	**25 (4.9)**
Sample thickness	5 (1.2)	0 (0.0)	5 (1.0)
Unspecified	4 (0.9)	1 (1.1)	5 (1.0)
Calibration vortix mixer	1 (0.2)	2 (2.2)	3 (0.6)
Automated IHC platform	3 (0.7)	N/A	3 (0.6)
Fluorescence microscope	2 (0.5)	N/A	2 (0.4)
Section of sample lost during pre-processing	1 (0.2)	0 (0.0)	1 (0.2)
Sample damaged by machine	1 (0.2)	0 (0.0)	1 (0.2)
Pressure cooker	1 (0.2)	0 (0.0)	1 (0.2)
Contamination due to sealing of tubes problem	1 (0.2)	0 (0.0)	1 (0.2)
Coverslipping workstation	1 (0.2)	0 (0.0)	1 (0.2)
Inadequate DNA template	0 (0.0)	1 (1.1)	1 (0.2)
Next-generation sequencing library preparation	0 (0.0)	1 (1.1)	1 (0.2)
**Other:** Any issue not related to the categories mentioned above	**0 (0.0)**	**1 (1.1)**	**1 (0.2)**
Abbreviations: *ALK*: ALK receptor tyrosine kinase, EQA: External Quality Assessment, FISH: fluorescence in-situ hybridization, IHC: immunohistochemistry, mCRC: metastatic colorectal cancer, N/A: not applicable as technique not offered during the EQA schemes, NSCLC: Non-small cell lung cancer, PCR: polymerase-chain reaction, SNP: single nucleotide polymorphism.			
