# Supplementary material for: Causes behind error rates for predictive biomarker testing: the utility of sending post-EQA surveys
Source: Virchows Arch. 2020 Nov 23;478(5):995–1006. doi: 10.1007/s00428-020-02966-7 (PMC8099794; doi:10.1007/s00428-020-02966-7)
Supplement: Supplementary file 4 — (PDF 339 kb) [file 428_2020_2966_MOESM4_ESM.pdf]

Title	Causes behind error rates for predictive biomarker testing: the utility of sending post-EQA surveys.
Journal	Virchows Archiv
Authors	Keppens Cleo, Schuuring Ed, Dequeker MC Elisabeth
Correspondence	Prof. Dr. Elisabeth Dequeker, University of Leuven,<br>Department of Public Health and Primary Care,<br>Biomedical Quality Assurance Research Unit,<br>Kapucijnenvoer 35d, Box 7001, Leuven 3000, Belgium.<br>Tel: +3216 345881, E-mail: els.dequeker@kuleuven.be
File	Supplemental Figure 1: Corrective/preventive actions undertaken to manage deviating EQA results for the different error time points and causes.

Supplemental Figure 1: Corrective/preventive actions undertaken to manage deviating EQA results for the different error time points and causes.

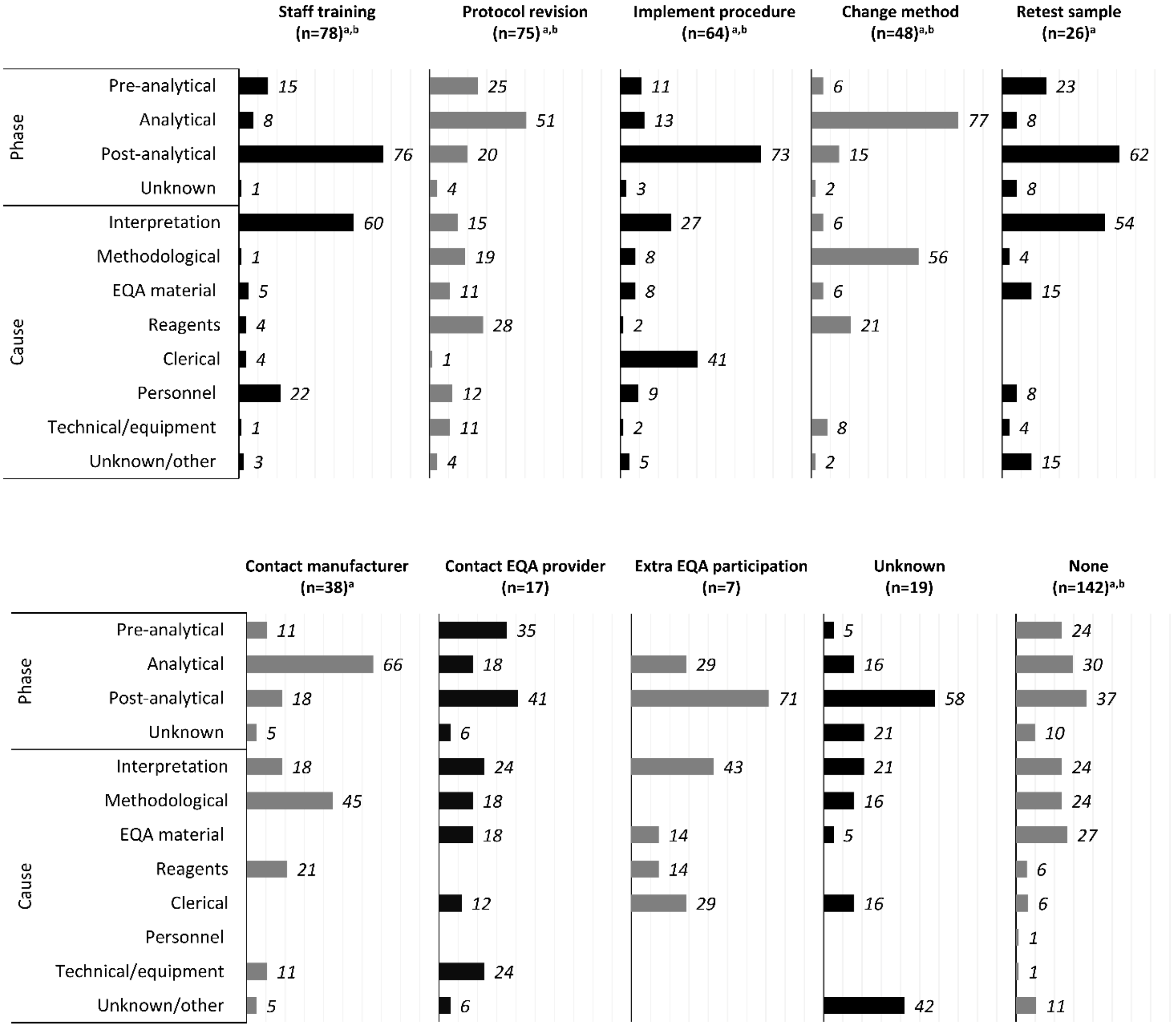

Bars and numbers in italic represent the percentage of the action reported to resolve specific problem.

Start and endpoints of phases in this study were defined based on definitions in ISO15189 (clauses 3.14 and 3.15) [15]. The pre-analytical phase was communicated in the survey as the time from sample reception until selection and estimation of the neoplastic cell percentage during pathologist review (for variant analysis) and until sample pre-treatment (for FISH or IHC). The analytical phase started from DNA extraction (if applicable) and the actual biomarker test, i.e. all steps of mutation analysis, gene rearrangement, or IHC analysis according to the pre-determined protocol. The post-analytical phase occurred between the readout of the analytical results (interpretation of mutation analysis curves, of the staining intensity/pattern, or reading of the split/single FISH nuclei), and reporting of the results, in this case when entering the results in the electronic EQA datasheets

A more detailed description of the definitions for the different error causes is given in **Supplemental Data 3**.

Statistics were calculated by proportional odds models with generalized estimating equations (GEE).

a. Significant ($p<0.05$) difference depending on problem phase (global test).

b. Significant ($p<0.05$) difference depending on problem cause (global test).

Abbreviations: EQA: External Quality Assessment.
